# Cbl Enforces Vav1 Dependence and a Restricted Pathway of T Cell Development

**DOI:** 10.1371/journal.pone.0018542

**Published:** 2011-04-07

**Authors:** Jeffrey Chiang, Richard J. Hodes

**Affiliations:** 1 Experimental Immunology Branch, National Cancer Institute, National Institutes of Health, Bethesda, Maryland, United States of America; 2 National Institute on Aging, National Institutes of Health, Bethesda, Maryland, United States of America; New York University, United States of America

## Abstract

Extensive studies of pre-TCR- and TCR-dependent signaling have led to characterization of a pathway deemed essential for efficient T cell development, and comprised of a cascade of sequential events involving phosphorylation of Lck and ZAP-70, followed by phosphorylation of LAT and SLP-76, and subsequent additional downstream events. Of interest, however, reports from our lab as well as others have indicated that the requirements for ZAP-70, LAT, and SLP-76 are partially reversed by inactivation of c-Cbl (Cbl), an E3 ubiquitin ligase that targets multiple molecules for ubiquitination and degradation. Analysis of signaling events in these Cbl knockout models, including the recently reported analysis of SLP-76 transgenes defective in interaction with Vav1, suggested that activation of Vav1 might be a critical event in alternative pathways of T cell development. To extend the analysis of signaling requirements for thymic development, we have therefore assessed the effect of Cbl inactivation on the T cell developmental defects that occur in Vav1-deficient mice. The defects in Vav1-deficient thymic development, including a marked defect in DN3-DN4 transition, were completely reversed by Cbl inactivation, accompanied by enhanced phosphorylation of PLC-γ1 and ERKs in response to pre-TCR/TCR cross-linking of Vav1^-/-^Cbl^-/-^ DP thymocytes. Taken together, these results suggest a substantially modified paradigm for pre-TCR/TCR signaling and T cell development. The observed consensus pathways of T cell development, including requirements for ZAP-70, LAT, SLP-76, and Vav1, appear to reflect the restriction by Cbl of an otherwise much broader set of molecular pathways capable of mediating T cell development.

## Introduction

The signal transduction pathways underlying T cell development and activation have been intensively studied. It has been demonstrated that pre-TCR/TCR stimulation results in activation of a signaling cascade initiated by phosphorylation and activation of TCR-ζ, Lck, and ZAP-70, which in turn phosphorylate downstream targets including LAT and SLP-76 [1], [2]. LAT and SLP-76 then activate downstream molecules such as Grb2, GADS, Vav1, ITK and PLC-γ [3], [4]. This cascade represents a widely cited current model of signaling requirements for T cell development. Evidence supporting the requirement for specific elements of this cascade has included biochemical analysis of pre-TCR/TCR signaling as well as compelling data derived from genetic manipulation to knock out or inactivate the genes encoding components of this pathway. Thus, inactivation of individual genes such as those encoding Lck, ZAP-70, LAT, SLP-76, or Vav1 results in severe compromise in thymic T cell development. Of interest, however, our work and that of other laboratories has suggested that the requirements for components of this canonical TCR signaling pathway may not be absolute, and that observed requirements may reflect, at least in part, events that limit the ability of alternative molecular mechanisms to support pre-TCR/TCR-driven T cell development.

Among the molecules that have been demonstrated to have a strong negative regulatory influence on TCR signaling is the Cbl family of proteins [5]. Three members of the Cbl family, c-Cbl (Cbl), Cbl-b and Cbl-3, are expressed in mammalian cells. All Cbl members contain a highly conserved N-terminal region containing a TKB (tyrosine kinase binding) domain and a RING finger domain that has enzymatic E3 ubiquitin ligase activity which mediates inhibition of TCR signal transduction. Only Cbl and Cbl-b have proline-rich region and UBA (ubiquitin associated) domains [5], [6]. Cbl proteins interact with multiple signaling molecules including Lck, Vav, TCR-ζ, PLC- γ1, and Grb2[5], [7]. Cbl has been identified as the most critical member of the Cbl family for regulation of thymic development. The Cbl knockout mouse model revealed that Cbl down-regulates, either directly or indirectly, the activation of TCR- ζ, Lck, ZAP-70, LAT and SLP-76, and influences the process of positive selection during T cell development [8], [9].

The function of Cbl has been further analyzed by assessing the effect of Cbl inactivation on the T cell developmental defects that occur consequent to a number of genetic manipulations. Cbl inactivation was found to partially rescue the developmental defect in ZAP-70 deficient mice[10], where thymocyte development is blocked at the DP stage, with very few SP thymocytes or peripheral T cells [11]. Cbl inactivation also partially rescued T cell development in LAT and SLP-76 deficient mice [12] which have no detectable DP or SP thymocytes or peripheral T cells [13], [14], [15]. These observations indicate that Cbl mediates the requirements for LAT, SLP-76 and ZAP-70 in thymocyte development by repressing signaling events that are capable of supporting T cell differentiation through a pathway or pathways that are independent of LAT, SLP-76, or ZAP-70. Most recently, an analysis of Cbl inactivation effects on the T cell developmental defects observed in SLP-76 mutant mice suggested that activation of Vav1 might be a critical event in rescue of alternative pathways of development[16]. To further address the signaling requirements for T cell development, we therefore asked whether T cell development in Cbl-deficient mice is dependent upon Vav1 function. Vav1-deficient mice exhibit substantial defects in T cell development, marked by a block at the DN3 stage and dramatically reduced activities of ERKs and PLC- γ1[17], [18], critical downstream mediators of Vav signaling in response to TCR stimulation. We demonstrated that the requirement for Vav1 is completely eliminated in Vav1^-/-^Cbl^-/-^ mice, with full normalization of T cell development.

## Materials and Methods

### Mice

c-Cbl knockout (Cbl^-/-^) and Vav1 knockout (Vav1^-/-^) mice were previously described[8], [19]. All animals were housed at Bioqual (Rockville, MD).

### Antibodies

Anti-mouse CD3ε (2C11), anti-CD28 (4F10), anti-CD4-PE, anti-CD8-FITC, anti-CD25-biotin, anti-leu4-biotin, anti-CD5-biotin, anti-HSA-biotin, and anti-TCR-β (H57)-biotin monoclonal antibodies were purchased from Pharmingen (San Diego, CA). Anti-phospho-tyrosine (4G10) and anti-PLC- γ1were purchased from Upstate (Lake Placid, NY). Anti-ERKs, anti-pY(416)Lck, anti-Lck and anti-phospho-ERKs polyclonal antibodies were purchased from Cell Signaling (Beverly, MA). Anti-Vav2 polyclonal goat antibody was purchased from Abcom (Cambridge, MA).

### Western blot analysis

Thymocytes were incubated with 5 µg/ml of biotin-conjugated anti-CD3 for 30 min, then stimulated with 10 µg/ml of streptavidin for the indicated time. The stimulated cells were lysed in buffer containing 50 µm Tris (pH 7.4), 150 µm NaCl, 1 mM Na2VO4, 1%NP-40 and protease inhibitor cocktail. Protein lysates were used for biochemical analysis[16].

### T cell proliferation

T cells were prepared from spleen and lymph nodes with MAC cell isolation kit (Miltenyi Biotech, Auburn, CA). One million cells per ml were plated in dishes coated with 1 µg/ml anti-mouse CD3 antibody and 1 µg/ml anti-mouse CD28 antibody after labeling of T cells with 1 µm CarboxyFluorescein Succinimidyl Ester (CFSE). Flow cytometric analysis was performed after two-day culture at 37°C[20].

## Results

### Cbl inactivation reverses the deficiency of T cell development in Vav1 knockout mice

**Figure 1:**
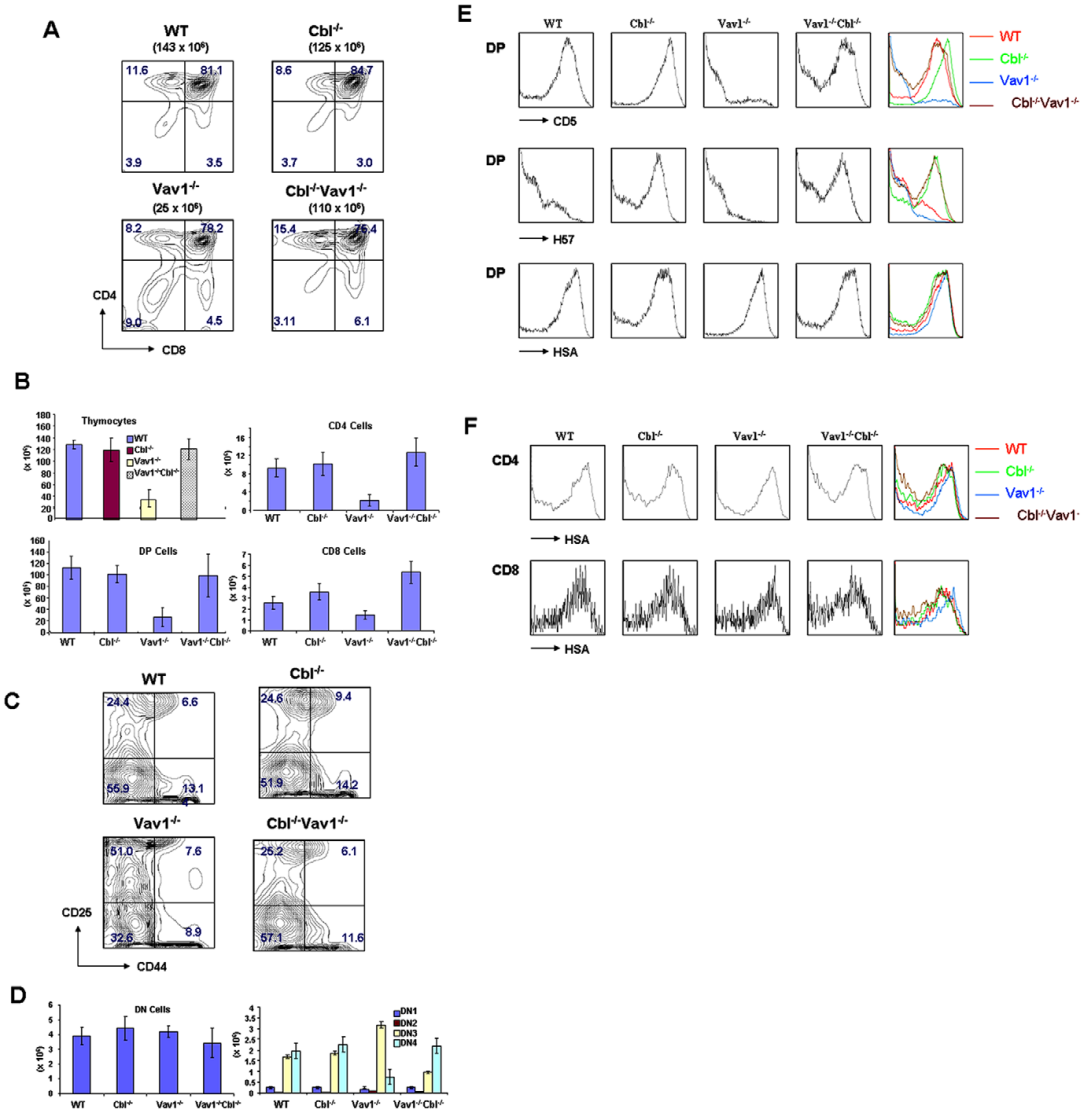
Cbl inactivation reverses the T cell development deficiency of Vav1 knockout mice. A) Thymocytes from WT (wild-type), Cbl^-/-^, Vav1^-/-^ and Cbl^-/-^Vav1^-/-^ mice were stained with CD4 and CD8, and assessed by flow cytometry. Genotypes of mice used in the experiment and total thymocytes of each mouse are shown above plots. Numbers in quadrants indicate the frequency of DN (CD4^-^CD8^-^), DP (CD4^+^CD8^+^), CD4 (CD4^+^CD8^-^), and CD8 (CD4^-^CD8^+^) cells. Results shown are representative of three or more experiments. B) Bar graph representing the mean total cellularity of thymocytes in WT (n = 5), Cbl^-/-^(n = 3), Vav1^-/-^ (n = 5) and Cbl^-/-^Vav1 (n = 5) mice, and the mean numbers of DN, DP, CD4^+^CD8^-^ (CD4) and CD4^-^CD8^+^ (CD8) thymocytes in WT (n = 5), Cbl^-/-^(n = 3), Vav1^-/-^ (n = 5) and Cbl^-/-^Vav1^-/-^ (n = 5) mice. C) Thymocytes from WT, Cbl^-/-^, Vav1^-/-^ and Cbl^-/-^Vav1^-/-^ mice were stained with CD25 and CD44, gated to exclude CD4^+^/CD8^+^/NK1.1^+^/Mac-1^+^/Gr1^+^/CD3^+^/B220^+^ cells, and assessed by flow cytometry. Genotypes of mice are shown above plots. Numbers in quadrants indicate the frequency of DN1 (CD25^-^CD44^+^), DN2 (CD25^+^CD44^+^), DN3 (CD25^+^CD44^-^) and DN4 (CD25^-^CD44^-^) thymocytes. Results shown are representative of three or more experiments. D) Bar graph representing the mean numbers of DN1, DN2, DN3, and DN4 thymocytes in WT (n = 5), Cbl^-/-^(n = 3), Vav1^-/-^ (n = 5) and Cbl^-/-^Vav1^-/-^ (n = 5) mice. E) Expression of cell surface molecules (CD5, HSA and H57 indicated at the bottom of histograms) on DP WT, Cbl^-/-^, Vav1^-/-^ and Cbl^-/-^Vav1^-/-^ thymocytes was assessed by flow cytometry. F) Expression of HSA on CD4 and CD8 cells of WT, Cbl^-/-^, Vav1^-/-^ and Cbl^-/-^Vav1^-/-^ thymocytes was assessed by flow cytometry.

The total number of thymocytes as well as the total number and percentage of SP and DP thymocytes are significantly decreased in Vav1^-/-^ mice in comparison with age-matched wild type mice[19]. In addition, the percentage of DN3 cells is increased in Vav1^-/-^ thymus and the percentage of DN4 cells is decreased, consistent with a block in differentiation at the DN3-DN4 transition or pre-TCR checkpoint. To test the effect of Cbl inactivation on these defects, Cbl^-/-^Vav1^-/-^ mice were generated and thymocytes analyzed by flow cytometry. Fig. 1 illustrates the defects in Vav1^-/-^ thymocytes observed in the present study, consistent with previous reports[19]. Strikingly, thymic development of Vav1^-/-^ mice was fully rescued by inactivation of Cbl. Cbl^-/-^Vav1^-/-^ mice had normal total thymocyte numbers, and normal percentages of DN, DP and SP cells, in comparison with WT mice (Fig. 1A, B). The block at the DN3 stage of Vav1^-/-^ thymocyte development was also reversed by the deletion of Cbl (Fig. 1C and Fig. 1D summary and statistical analysis).

Additional abnormalities seen in Vav1-deficient thymocytes include decreased expression of TCR-β and a marked reduction in expression of CD5, an activation marker that reflects strength of in vivo signaling of DP cells. The decreased expression of both TCR-β and CD5 was reversed in Cbl^-/-^Vav1^-/-^ DP cells (Fig. 1E). HSA, a marker which is progressively decreased in cell surface expression during maturation of thymocytes, remained abnormally elevated in Vav1-deficient DP and SP thymocytes, suggesting defective terminal maturation, and was restored to normal levels by the deletion of Cbl (Fig. 1E, F).

### Cbl inactivation does not rescue the phenotype of peripheral T cells

**Figure 2:**
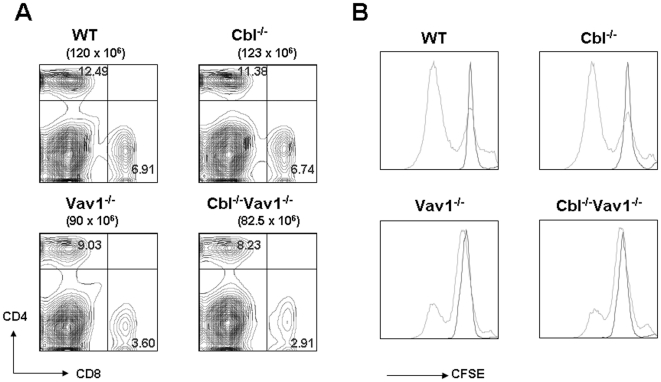
Cbl inactivation does not rescue the phenotype of Vav1-deficient peripheral T cells. A). Splenocytes from WT, Cbl^-/-^, Vav1^-/-^ and Cbl^-/-^Vav1^-/-^ mice were stained with CD4 and CD8, and assessed by flow cytometry. Genotypes of mice used in the experiment and total splenocytes of each mouse are shown above plots. Numbers in quadrants indicate the frequency of CD4 and CD8 cells. Results shown are representative of five or more experiments. B). Mature T cells were isolated from WT, Cbl^-/-^, Vav1^-/-^ and Cbl^-/-^Vav1^-/-^ spleens and lymph nodes. The purified T cells were stained with CFSE, and plated on tissue culture plates coated with 1 µg/ml anti-mouse CD3 antibody and 1 µg/ml ant-mouse CD28 antibody. Cell proliferation was assessed by flow cytometric measurement of CFSE dilution.

Vav1^-/-^ splenocytes have significantly reduced percentages and total cell numbers of CD4 and CD8 T cells. In contrast to the observed reversal of thymic phenotypes by Cbl inactivation, these peripheral abnormalities were not reversed in Cbl^-/-^ Vav1^-/-^ mice (Fig. 2A). The defective T cell proliferative response of Vav1^-/-^ CD4 or CD8 cells to TCR cross-linking was also not reversed in Cbl^-/-^Vav1^-/-^ peripheral T cells (Fig. 2B).

### Normally Vav1-dependent signaling events are enhanced in Cbl^-/-^Vav1^-/-^ thymocytes

**Figure 3:**
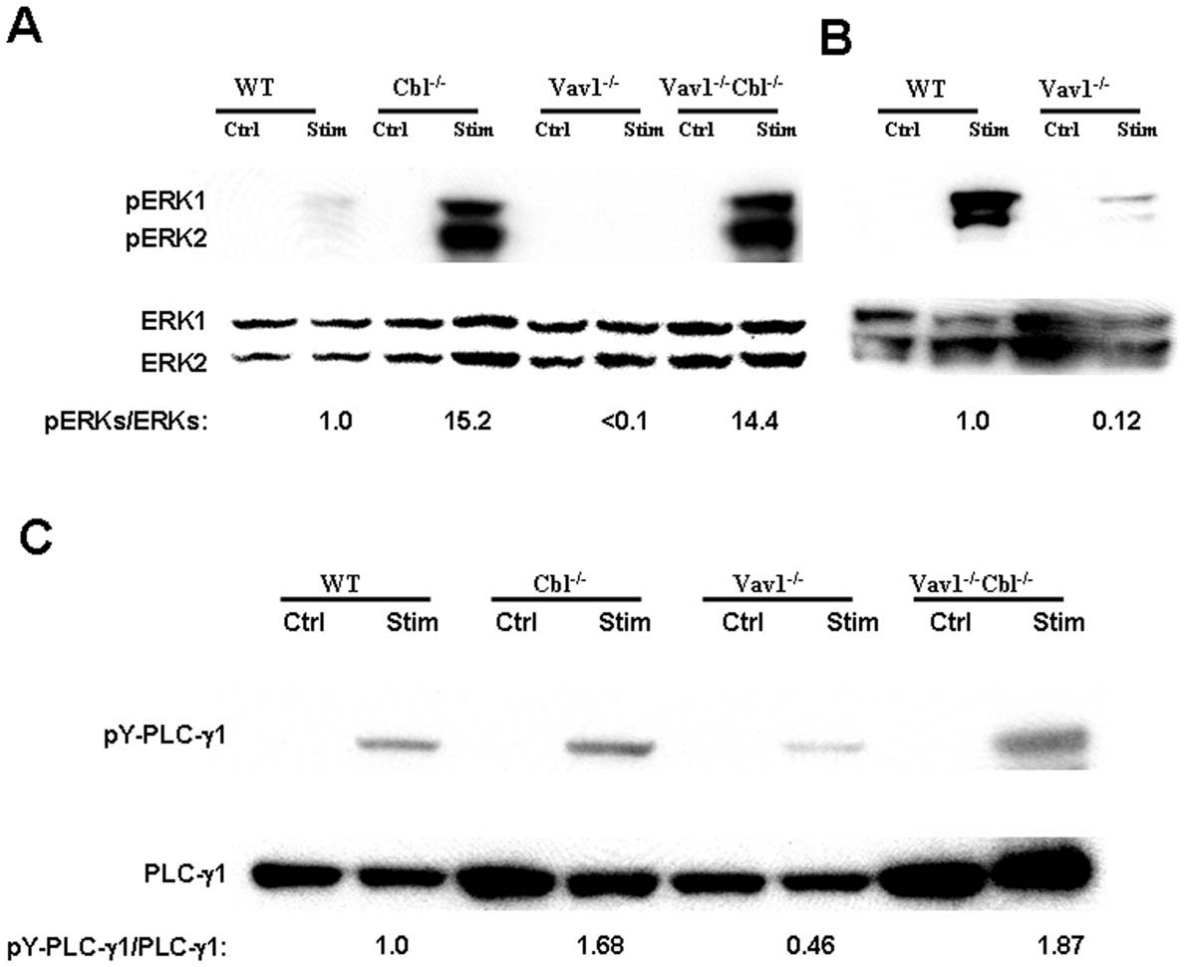
Phosphorylation of normally Vav1-dependent signaling molecules is enhanced in Cbl^-/-^Vav1^-/-^ thymocytes. DP thymocytes were sorted from WT, Cbl^-/-^, Vav1^-/-^ and Cbl^-/-^Vav1^-/-^ mice by flow cytometry, cultured without stimulation (Ctrl) or with anit-CD3 Ab (Stim) for 2 minutes at 37°C. and protein lysates prepared. A) Protein lysates were immunoprecipitated (IP) with anti-PLC γ1 and immunoblotted (IB) with anti-phosphotyrosine (4G10) (upper panel) or anti-PLC γ1 (lower panels). The results presented are representative of 4 experiments. B) Analysis as in Panel A, but with samples run and exposed to allow visualization and comparison of WT and Vav^-/-^ DP thymocytes. C) Protein lysates were immunoblotted (IB) with anti-phospho-ERKs (upper panel) or anti-ERK (lower panel). The results presented are representative of 4 experiments.

It has been reported that Vav1 inactivation results in reduced phosphorylation and activation of ERKs and PLC-γ1 in thymocytes[17], [18]. To determine whether Cbl regulates these normally Vav-dependent signaling events, thymocytes from Cbl^-/-^Vav1^-/-^ or control mice were stimulated with cross-linking anti-CD3 antibody, and (Thr202/Tyr204) phosphorylation of ERKs and tyrosine-phosphorylation of PLC- γ1 were analyzed by immunoblotting. Phosphorylation of ERK1 and ERK2 was dramatically reduced in Vav1^-/-^ thymocytes relative to wild-type controls (Fig. 3A,B), consistent with previous publication[17], [18]. The phosphorylation of ERK1 and ERK2 in Cbl^-/-^Vav1^-/-^ thymocytes was substantially increased in comparison with Vav1^-/-^ cells, and was similar to that of Cbl^-/-^ cells whose phosphorylation level was dramatically higher than wild type thymocytes. Tyrosine phosphorylation of PLC- γ1 in Vav1^-/-^ thymocytes was similarly decreased in comparison with wild type thymocytes. The tyrosine phosphorylation of PLC- γ1 in Cbl^-/-^ as well as Cbl^-/-^Vav1^-/-^ thymocytes in response to TCR stimulation was greatly increased in comparison with that of Vav1^-/-^ and wild type cells (Fig. 3C). The deletion of Cbl thus reversed the defects in phosphorylation of ERKs and PLC- γ1 in Vav1^-/-^ thymocytes and enables Cbl^-/-^Vav1^-/-^ thymocytes to efficiently generate TCR-activated signaling events that are normally dependent upon Vav1.

### Multiple–Vav1- independent signaling events are increased in Cbl^-/-^Vav1^-/-^ thymocytes

**Figure 4:**
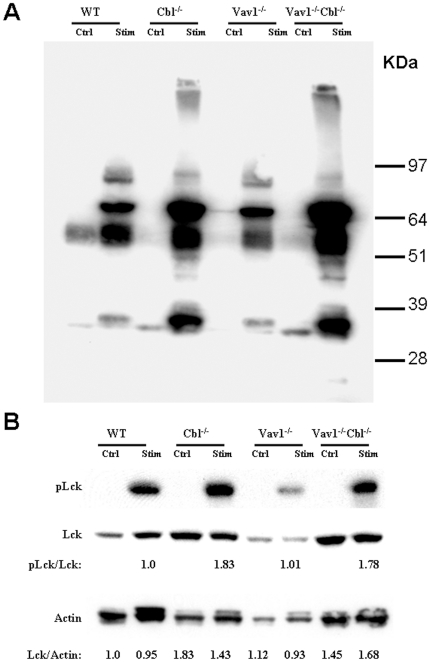
Multiple Vav1- independent signaling events are increased in Cbl^-/-^Vav1^-/-^ thymocytes. DP thymocytes were sorted from WT, Cbl^-/-^, Vav1^-/-^ and Cbl^-/-^Vav1^-/-^ mice by flow cytometry, cultured without stimulation or with anit-CD3 Ab for 2 minutes at 37°C, and protein lysates prepared. A) Protein lysates were immunoblotted (IB) with anti-phosphotyrosine Ab (4G10). The results presented are representative of 4 experiments. B) Protein lysates were immunoblotted (IB) with anti phospho(P)-lck (upper panel), anti-Lck (middle panel), or anti-actin antibody (Lower panel). The results presented are representative of 3 experiments.

The effect of Cbl inactivation in rescuing the development defect in Vav1^-/-^ thymocytes might result from the effects identified above, through activation of events that are normally mediated by Vav1. However, it is also possible that Cbl inactivation functions through effects on signaling events distinct from those mediated by Vav1. We therefore measured total protein tyrosine phosphorylation of Cbl^-/-^Vav1^-/-^ thymocytes to broadly assess the effect of Cbl inactivation on candidate signaling molecules activated by TCR cross-linking in these cells. Total protein tyrosine phosphorylation of Cbl^-/-^Vav1^-/-^ thymocytes was significantly increased in comparison with that of Vav1^-/-^ or wild type thymocytes in response to TCR stimulation, as reflected by increased density of multiple discrete bands (Fig. 4A). Multiple bands representing proteins that are hyperphosphorylated in Cbl^-/-^Vav1^-/-^ thymocytes were equivalent to the bands observed in singly deficient Cbl^-/-^ cells here and in previous reports[8], [9]. Many of these bands were not detectably different in wild type versus Vav1^-/-^ thymocytes, indicating that they are not normally Vav1-dependent.

Phosphorylation and consequent activation of Lck have been described as critical early events in TCR-mediated signaling. We determined phosphorylation of Lck in response to TCR stimulation, using antibody specific for phosphorylation of Y416 of Lck, a modification that has been specifically linked to activation of Lck kinase activity. Equivalent levels of Lck Y416 phosphorylation were observed in wild type and Vav1^-/-^ thymocytes in response to TCR stimulation, indicating that this Lck phosphorylation response is not Vav1-dependent, in contrast to effects on ERKs and PLC- γ1. The level of phosphorylation of Lck in Cbl^-/-^ thymocytes was higher than in wild type cells, consistent with previous reports[16]. Lck Y416 phosphorylation in Cbl^-/-^Vav1^-/-^ thymocytes was also elevated and was equal to that in Cbl^-/-^ thymocytes and higher than that of wild type and Vav1^-/-^ cells (Fig. 4B). The level of total Lck protein was increased in both Cbl^-/-^Vav1^-/-^ and Cbl^-/-^ thymocytes while total Lck in Vav1^-/-^ cells was similar to that in WT cells (Fig. 4B). Cbl inactivation, which rescues the developmental defect in Vav1^-/-^ thymocytes, thus enhanced activities of multiple signaling molecules, including the early TCR-mediated signal molecule Lck, in addition to enhancing normally Vav1-dependent downstream events.

## Discussion

A current accepted model for pre-TCR and TCR signal transduction during T cell development, as well as for TCR-mediated activation of mature T cells, involves critical early roles for Lck, ZAP-70, LAT, and SLP-76, which in turn transduce signals to downstream molecules including PLC- γ1, Vav, and ERKs[1], [2]. Inactivating Lck, ZAP-70, LAT, or SLP-76 results in severely defective thymic development[11], [13], [15]. However, it has been demonstrated that deletion of Cbl can partially rescue the defects in T cell development observed in ZAP-70, LAT or SLP-76 deficient mice[10], [12], [21], indicating the existence of an alternative signaling pathway normally repressed by Cbl, that is independent of canonical requirements for ZAP-70, LAT, and SLP-76. We recently reported findings suggesting that Vav1 is a critical factor in this alternative signaling pathway for T cell development. SLP-76^-/-^Cbl^-/-^ mice reconstituted with a mutant SLP76 transgene (Y3F) which is unable to bind Vav[16], [22],exhibit severe defects of thymic development, coupled with impaired Vav1 phosphorylation in response to TCR cross-linking [16]. Notably, these defects in both T cell development and Vav1 phosphorylation were completely reversed by Cbl inactivation. Cbl inactivation might thus restore thymic differentiation in SLP-76^-/-^Y3F mice by allowing Vav1 activation and permitting T cell development in absence of normally essential upstream events. Consistent with this possibility, biochemical studies have revealed that Cbl protein can directly interact with and degrade Vav1 through its E3 ligase enzymatic activity[23]. In the present study, we generated and analyzed Cbl^-/-^Vav1^-/-^ mice to determine whether rescue of thymic differentiation by Cbl inactivation is in fact dependent on Vav1.

We observed normal numbers of thymocytes, and normal percentages and numbers of DN, DP and SP cells in Cbl^-/-^Vav1^-/-^ thymus, reversing the defects that are seen in Vav1^-/-^ mice (Fig. 1A, B). We also found that Cbl^-/-^Vav1^-/-^ mice had normal development through the DN stage, reversing the block at DN3 that occurs in Vav1^-/-^ mice (Fig. 1C, D). Phenotypic alterations, marked by reduced expression of TCR and CD5 in Vav1^-/-^ DP thymocytes, and elevated HSA in SP thymocytes, were also reversed in Cbl^-/-^Vav1^-/-^ cells (Fig. 1E). Inactivation of Cbl therefore results in the restoration of normal T cell development in Vav1^-/-^ mice, demonstrating that the alternative pathway enabled by Cbl inactivation is independent of Vav1. To reveal what signaling events resulting from Cbl inactivation might mediate the reversal of defects in Vav1^-/-^ mice, phosphorylation events were analyzed in Cbl^-/-^Vav1^-/-^ thymocytes. These studies indicated that TCR-induced phosphorylation of ERKs and PLC- γ1, events that are normally Vav1-dependent, were prominently enhanced by Cbl inactivation, suggesting that Cbl normally modulates signaling molecules, such as critical ERKs and PLC- γ1, which are downstream of Vav1 signal transduction. It has in fact been reported that Cbl can directly interact with PLC- γ1 and inhibit its activity[24], and can interact with and modulate the function of Grb2 that in turn activates ERKs[25], [26]. Our attempts to directly test the role of other Vav family members in the normalized development observed in Cbl^-/-^Vav1^-/-^ thymocytes were unsuccessful due to a failure in extensive efforts to breed Cbl^-/-^Vav1/2/3^-/-^ mice. We observed that phosphorylation of Vav2 was significantly increased in Cbl^-/-^Vav1^-/-^ thymocytes in response to TCR stimulation, while total Vav2 protein level was not affected (Figure S1). However, any enhanced activities of Vav2 and Vav3 might not activate the same signal molecules as those whose functions were lost in Vav1^-/-^ cells. Furthermore, deficiency in Vav1 resulted in impaired T cell development while normal T cell development was seen in Vav2/3 double knockout mice[27]. In addition to enhancing Vav1-dependent signaling events such as phosphorylation of ERKs and PLC- γ1, Cbl inactivation resulted in enhanced Vav1-independent TCR-induced tyrosine phosphorylation of multiple proteins in Cbl^-/-^Vav1^-/-^ thymocytes (Fig. 4A). Notably, Lck phosphorylation, regarded as a very proximal event in TCR signal transduction, was not affected by Vav1 inactivation but was markedly increased in Cbl^-/-^Vav1^-/-^ thymocytes in comparison with wild-type cells (Fig. 4B). The signaling events amplified in Cbl^-/-^Vav1^-/-^ thymocytes thus include both Vav1-dependent and Vav1-independent events that could contribute to rescued thymocyte development.

Although Cbl^-/-^Vav1^-/-^ mice were shown here to have normal T cell development, these mice had persistent defects in homeostasis and proliferation of peripheral T cells, not differing from those seen in Vav1^-/-^ (Fig. 2A, B). It has been reported that the expression of Cbl is substantially higher in thymus than in peripheral T cells, while expression of Cbl-b is higher in peripheral T cells than in thymocytes[28]. It is therefore possible that the relatively high expression of Cbl-b in peripheral T cells prevents inactivation of Cbl from reversing the defective phenotypes of peripheral T cells in Vav1^-/-^ mice. Consistent with this possibility, Cbl-b and Vav1 double knockout mice have been studied by Krawczyk et al[29], who reported that Cbl-b inactivation did not reverse the defects in thymic development in Vav1^-/-^ mice but did reverse defective proliferation of peripheral T cells. Therefore, the interaction between Cbl or Cbl-b with Vav1 protein might have different impact in T cell differentiation and in T cell activation.

In summary, Cbl inactivation rescued defective T cell development in Vav1^-/-^ mice, indicating that Cbl normally represses signaling events that can mediate thymic differentiation in the absence of Vav1. Thus, Cbl inactivation dramatically alters the signaling requirements for thymic T cell differentiation, revealing effective pathways that are independent of Vav1 as well as ZAP-70, LAT, and SLP-76. These findings suggest a substantially modified paradigm for pre-TCR/TCR signaling and T cell development. There may be a broad array of molecular interactions that are capable of transmitting pre-TCR or TCR engagement into events sufficient to drive differentiation and selection. The ability of these interactions to be functional is dependent upon kinetics of molecular interaction, driven by the abundance, survival, and trafficking of specific components. The observed requirement for specific molecules, including ZAP-70, LAT, SLP-76, and Vav1, in T cell development reflects conditions in which this diverse set of alternative pathways is severely limited by the effects of regulators, notably including Cbl. Cbl, through activities including its function as an E3 ubiquitin ligase, limits many of the molecular interactions and events that could otherwise drive T cell differentiation, and thus enforces the requirements observed in canonical pathways of pre-TCR/TCR signaling.

## Supporting Information

Figure S1 **Tyrosine phosphorylation of Vav2 in Cbl^-/-^Vav1^-/-^ thymocytes was increased in response to TCR stimulation.** Protein lysates were immunoprecipitated (IP) with anti-Vav2 and immunoblotted (IB) with anti-phosphotyrosine Ab (4G10) (upper panel) and anti-Vav1 Ab (second panel), or were immunoblotted (IB) with anti-Vav2 (third panel), or anti-actin antibody (Lower panel). The results presented are representative of 2 experiments. Tyrosine phosphorylation of Vav2 in Cbl^-/-^Vav1^-/-^ thymocytes was increased in comparison with Vav1^-/-^ or wild type cells in response to TCR stimulation, while the total protein level of Vav2 in Cbl^-/-^Vav1^-/-^ thymocytes was equal to that of Vav1^-/-^ or wild type thymocytes.
(TIF)
