# Supplementary figures and images for: Cbl Enforces Vav1 Dependence and a Restricted Pathway of T Cell Development
Source: PLoS One. 2011 Apr 7;6(4):e18542. doi: 10.1371/journal.pone.0018542 (PMC3072394; doi:10.1371/journal.pone.0018542)

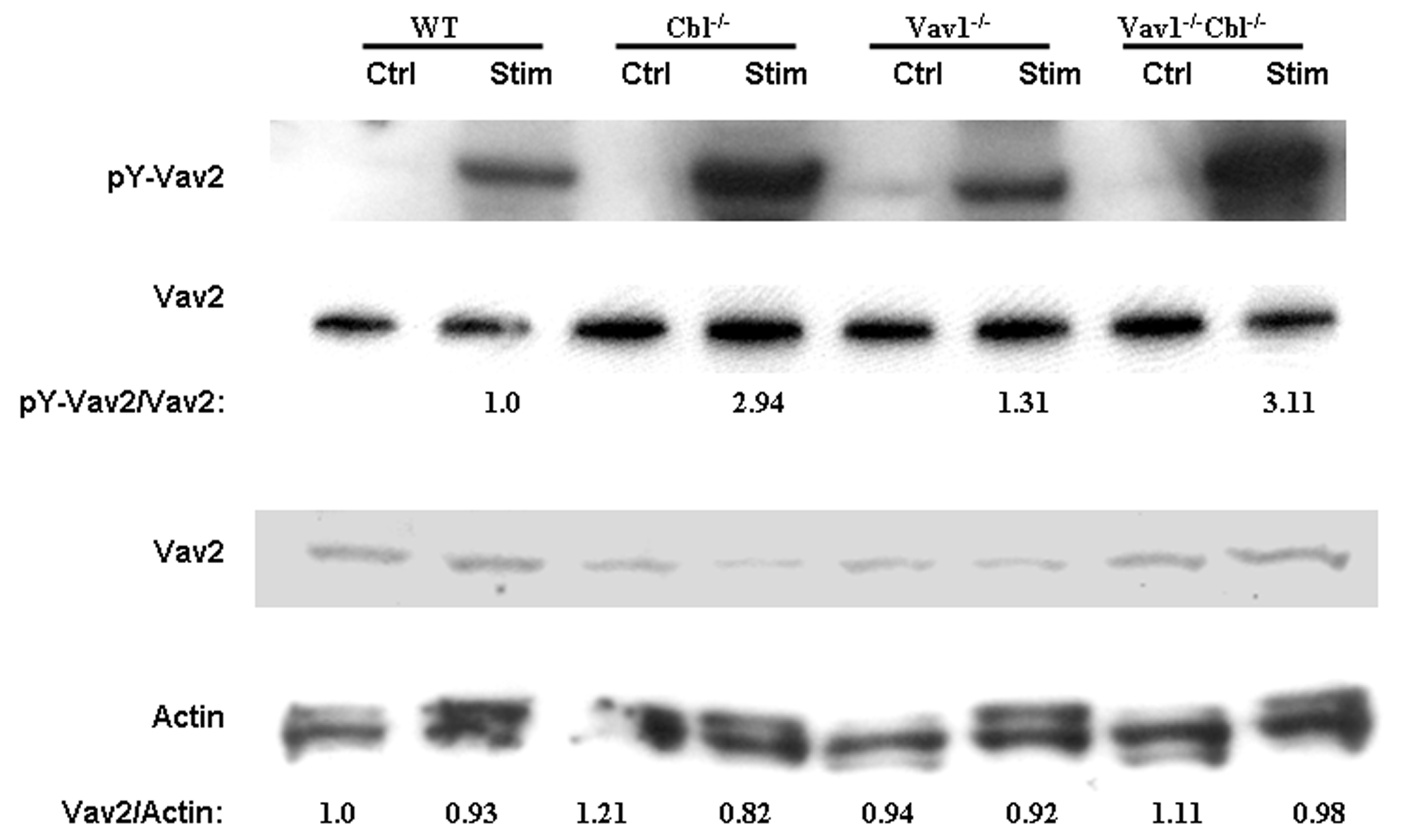

Supplement: Figure S1 — Tyrosine phosphorylation of Vav2 in Cbl-/-Vav1-/- thymocytes was increased in response to TCR stimulation. Protein lysates were immunoprecipitated (IP) with anti-Vav2 and immunoblotted (IB) with anti-phosphotyrosine Ab (4G10) (upper panel) and anti-Vav1 Ab (second panel), or were immunoblotted (IB) with anti-Vav2 (third panel), or anti-actin antibody (Lower panel). The results presented are representative of 2 experiments. Tyrosine phosphorylation of Vav2 in Cbl-/-Vav1-/- thymocytes was increased in comparison with Vav1-/- or wild type cells in response to TCR stimulation, while the total protein level of Vav2 in Cbl-/-Vav1-/- thymocytes was equal to that of Vav1-/- or wild type thymocytes. (TIF) [file pone.0018542.s001.tif]
